# Film bulk acoustic resonators integrated on arbitrary substrates using a polymer support layer

**DOI:** 10.1038/srep09510

**Published:** 2015-03-31

**Authors:** Guohao Chen, Xinru Zhao, Xiaozhi Wang, Hao Jin, Shijian Li, Shurong Dong, A. J. Flewitt, W. I. Milne, J. K. Luo

**Affiliations:** 1 Department of Information Science and Electronic Engineering, Zhejiang University and Cyrus Tang Center for Sensor Materials and Applications, Zhejiang University, Hangzhou 310027, China; 2 College of Computer Science, Zhejiang University, Hangzhou. 310027, China; 3 Electrical Engineering Division, University of Cambridge, JJ Thomson Avenue, Cambridge, CB3 0FA U.K.; 4 Institute of Renewable Energy and Environmental Technologies, Bolton University, Deane Road, Bolton BL3 5AB, UK

## Abstract

The film bulk acoustic resonator (FBAR) is a widely-used MEMS device which can be used as a filter, or as a gravimetric sensor for biochemical or physical sensing. Current device architectures require the use of an acoustic mirror or a freestanding membrane and are fabricated as discrete components. A new architecture is demonstrated which permits fabrication and integration of FBARs on arbitrary substrates. Wave confinement is achieved by fabricating the resonator on a polyimide support layer. Results show when the polymer thickness is greater than a critical value, d, the FBARs have similar performance to devices using alternative architectures. For ZnO FBARs operating at 1.3–2.2 GHz, d is ~9 μm, and the devices have a *Q*-factor of 470, comparable to 493 for the membrane architecture devices. The polymer support makes the resonators insensitive to the underlying substrate. Yields over 95% have been achieved on roughened silicon, copper and glass.

Advancements in microfabrication technology are enabling the development of a diversity of microelectromechanical systems (MEMS) devices, and these are being widely employed in the latest electronic devices, with the ubiquitous use of gyroscopes in smart phones and tablet computers being just two examples. Film bulk acoustic resonators (FBARs) are one such MEMS device with widespread applications as frequency regulators, filters and duplexers^1^,^2^ in communication electronics, as gravimetric sensors for physical (temperature^3^, pressure^4^, humidity^5^ and ultraviolet (UV) light^6^), chemical (organic gas^7^, toxic ions^8^ and explosives^9^), and biological (antigens and proteins^10^,^11^,^12^) sensing. FBAR sensors have extremely high sensitivity owing to their high resonant frequency (~1–3 GHz), high quality factor, *Q* (>250), and low base mass. Gravimetric FBAR sensors have been demonstrated with the capability to detect a mass as low as 10^−15^ g^11^,^13^.

FBARs have three basic device architectures: the back-trench membrane^3^, a membrane over an air-gap^14^ and the Bragg mirror reflector (also called the solid-mounted resonator)^15^. FBARs employ a film of piezoelectric (PE) material (*e.g*. zinc oxide (ZnO) or aluminum nitride (AlN)) with electrodes on either side to which a radio-frequency (RF) signal is applied. Reflections from the top and bottom surfaces of the piezoelectric film result in a resonant standing wave being produced at certain frequencies (*e.g.* where the film thickness equals half of one wavelength). The purpose of these architectures is to minimize acoustic losses from the piezoelectric film which would occur if the device is mounted directly on a simple substrate, such as silicon. This would greatly reduce the *Q*-factor and even supress the resonance. In the cases of the back-trench membrane and the membrane over an air-gap, the resonant structure is fabricated on a suspended membrane, typically SiO_2_^16^ or Si_3_N_4_^17^. These architectures require the removal of bulk material underneath by deep reactive ion etch (DRIE)^18^, or a sacrificial layer by wet and dry etching. These architectures often have very low yield due to a combination of the residual stresses in the suspended membrane and piezoelectric layer, and the lengthy etching processes. Bragg mirror structure FBARs are made on acoustic reflectors consisting of multiple layers of alternating low and high acoustic impedance films on a solid substrate without the need to remove the material underneath. Although this is the preferred structure owing to its robustness, the Bragg mirror requires long deposition times and precise control of the thickness for each layer down to a few nanometers (nm). It is thus not a cost-effective or high-throughput process, and it often requires a high temperature deposition in order to obtain high quality reflector layers, making the architecture incompatible with integration on CMOS or with other MEMS on the same substrate.

As a consequence, FBARs are commonly fabricated and used as discrete devices. There are great demands for the integration of FBARs with CMOS circuitry which would provide better control and signal processing^19^,^20^. Also integration with CMOS would reduce the influence of interconnects, bonding wires and other parasitic effects that may increase or change insertion loss^21^. Many requirements have to be fulfilled for successful device integration with CMOS. These include low thermal budget for the post-CMOS processes, good adhesion between layers, removal of sacrificial layers without damage to CMOS and MEMS materials, and selection of suitable electrodes and isolation materials that have to be compatible with all the processes and materials used in the fabrication of CMOS^22^. Existing FBAR architectures make this extremely challenging.

Here we present a new FBAR architecture which allows integration on almost any substrate (paper, glass and copper plates as well as roughened silicon are demonstrated) without compromising device performance. This is achieved by fabricating resonant structures on a polymer support layer with near-zero acoustic impedance that can confine the acoustic wave. Polyimide (PI) has been used in this work. The FBARs on these diverse substrates perform well with *Q*-factors up to 1000, which are comparable to or better than previous architectures, but with greatly simplified fabrication processes and with yield as high as 95% in laboratory conditions.

## Results

### Theoretical analysis and numerical modeling

**Figure 1:**
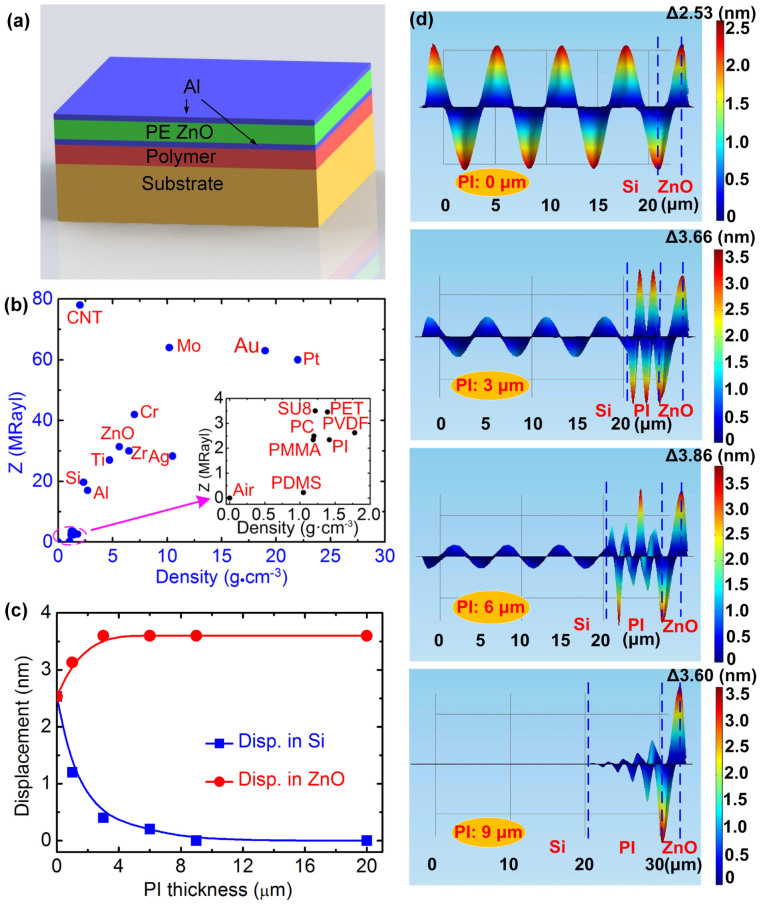
Analysis and modeling of the FBAR with a polymer support layer architecture. (a) Schematic of the PI-FBAR architecture, (b) Comparison of acoustic impedance of various materials used in FBAR fabrication, (c) Summary of the displacements in the PE layer and Si in the PI-FBAR architecture as a function of PI layer thickness, (d) Displacements in layers of the PI-FBAR architecture with varying PI thickness, clearly showing the wave is confined and diminishes within the PI layer when its thickness is greater than 9 μm, and no acoustic energy is transmitted to the elastic Si substrate. The thicknesses of the ZnO and Si layers are 2.0 and 20 μm, respectively for the modeling. (Note Figure 1d is not based on an exact 2D figure, but rather viewed from a wide angle towards to narrow one. For clearer view, please see the 3D figures in Figure S2b in SI).

Consider an FBAR consisting of a PE layer sandwiched by two metal electrodes, sitting on a polymer support layer on a substrate as shown in Figure 1a. For simplicity, it is assumed that the bottom electrode has similar properties to those of the PE layer, and it can be considered as part of the PE layer during the analysis as shown in Figure S1a in supplementary information (SI). Under RF signal excitation, standing (plane) waves are generated between the two electrodes. The reflectance, *R*, and transmittance, *T*, of the plane waves at an interface with the support layer are given by^23^,^24^
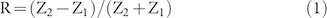

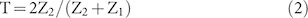
where Z*_i_* = *ρ_i_V_i_* represents the acoustic impedance, and *ρ_i_* and *V_i_* are the material density and acoustic velocity of the piezoelectric (*i* = 1) and support (*i* = 2) layers. When *Z*_1_ = *Z*_2_, *R* = 0 and *T = * 1, and there is total transmission of the waves into the bottom layer and no wave confinement within the PE layer; when *Z*_1_ ≫ *Z*_2_, *R* = 1 and *T* = 0, and there is total reflection, resulting in perfect confinement of the acoustic wave. For other cases, part of the acoustic wave is transmitted through the interface, and propagates with attenuation into the support and substrate layers. For FBARs, *R* = 1 would be the ideal case, and this is approached using the Bragg reflector or a membrane-over-air structure as air has zero acoustic impedance. However, equation 1 and 2 indicate that FBARs may be made on substrates with a polymer support layer which has extremely small *Z*_2_ compared with *Z*_1_. Figure 1b shows the acoustic impedance for materials normally used in fabricating FBAR devices^11^,^25^,^26^,^27^,^28^,^29^,^30^,^31^, indicating various polymer materials with *Z* close to that of air might be used as the support layer to fabricate FBARs directly on substrates without either the removal of the back material or use of the Bragg reflector.

Waves in a viscoelastic polymer are attenuated due to scattering and absorption as a result of energy loss due to viscosity and heat conduction. Based on Stoke's law for plane sine wave propagation along an *x*-direction in an isotropic and homogeneous medium^32^, the wave amplitude, *A*(*x*), decreases exponentially with distance *x*, and is given by
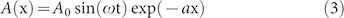
where *A_0_* is the initial amplitude and *α* is the attenuation coefficient which increases rapidly with the increase in angular frequency, *ω*, and temperature^33^,^34^. If the polymer support layer is thinner than the attenuation distance, acoustic waves may reach the interface of the polymer and the underlying substrate (*e.g.* Si). The low acoustic loss of the substrate leads to efficient transmission of the acoustic waves into the substrate bulk, and efficient removal of acoustic energy from the system, reducing the *Q*-factor of the resonator or leading to failure of the resonance completely. When the polymer is thicker than the attenuation distance, the acoustic waves disappear completely within the polymer layer with no acoustic energy transmitted to the Si substrate. The results imply that high performance FBARs can be made on solid substrates with a support layer of near-zero acoustic impedance and large attenuation coefficient. We may call such acoustic support layers the *near zero-index material* or *quasi acoustic metamaterials*.

To verify the theoretical model, we conducted a finite element analysis (FEA) for the architecture shown in Figure 1a for the case of a ZnO piezoelectric film on a polyimide support layer of varying thickness on a Si substrate. The ZnO PE layer is sandwiched by two aluminum (Al) electrodes on either side. Figures 1c and 1d show the displacement of the layers induced by RF excitation at resonance with the PI thickness as a variable. For the structure directly on the Si substrate, the acoustic wave transmits directly into the substrate with no obvious reflection at the interface of the PE and Si due to their similar acoustic impedance and elastic properties (see Figure 1b). There is little acoustic wave attenuation in Si as it is assumed that Si has perfect crystal structure with no acoustic scattering and absorption. When the FBAR is on a PI layer, the situation is very different. The displacement in the Si layer is large when the PI layer is thinner than the attenuation distance, and decreases as the PI layer becomes thicker, leading to an acoustic wave of reduced amplitude in the Si layer. The amplitude of the wave in the Si has reduced to near zero once the PI support layer thickness has reached ~9 μm.

The modeling results confirm that a polymer layer with low acoustic impedance and thickness greater than the attenuation length of the acoustic wave can be a support layer offering good acoustic isolation for FBARs from the substrate. The evolution of the acoustic waves in 2-dimensional (2D) and 3D with varying PI thickness in the FBAR structure is shown in Figure S2 in detail, and it clearly shows that the displacements in the Si layer decrease, while those in the PE layer increase with increase in the PI thickness, and become constant for a PI thickness above a critical value as summarized in Figure 1c.

### FBARs on a PI support layer on a Si substrate

**Figure 2:**
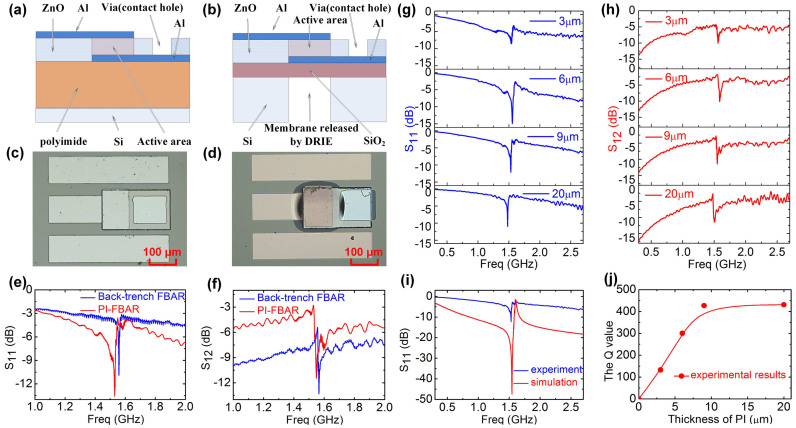
Comparison of the PI-FBAR and back-trench FBAR. (a) and (b) Schematics of the proposed PI-FBAR with a PI support layer and the back-trench FBAR; (c) & (d) Microscopy images of the fabricated PI-FBAR and back-trench FBAR; (e) & (f) Comparison of reflection (S_11_) and transmission spectra (S_12_) for the PI-FBAR (red lines) and trench FBAR (blue lines), showing they have similar performance with high signal amplitude and quality factor. (g) & (h) Reflection and transmission spectra of the fabricated PI-FBARs with the PI thickness as a variable. The amplitude of the resonance improves with increase in the PI thickness. (i) Comparison of the reflection spectra of the PI-FBARs obtained through FEA modeling and experimentally, showing a good agreement for the resonant frequency between them. (j) The *Q*-value of the resonators as a function of PI thickness. The *Q*-value increases initially with the increase in PI thickness, and then saturates when the PI thickness is greater than 9 μm.

FBARs with a PI support layer with varying thickness were fabricated to verify the model (hereafter we designate this type of device as the PI-FBAR). For comparison, back-trench etched FBARs were also fabricated at the same time with a 2 μm thickness thermal-grown SiO_2_ membrane. Figures 2a and 2b are 2D schematics of the PI-FBAR architecture and the back-trench FBAR on a Si substrate (3D schematics are shown in Figures S1b and S1c). All the devices have an active area of 100 × 200 μm. The substrate could be any solid material such as a copper plate, glass, metal foils or paper as shown later. A brief description of the process for the PI layer formation is as follows. A PI layer was formed on a Si substrate by spin-coating with the thickness varied from 2 to 20 μm. The PI layer was solidified by baking it at 240°C for 3 hr. The PI-FBARs were then fabricated by a three-mask process, which is the same as that used for the fabrication of the back-trench FBARs, but without the back-trench etching process stage – namely the bottom electrode formation, via formation and top electrode formation (details described in the Methods Section). Crystal structure characterization shows that the ZnO film is polycrystalline with highly-oriented columnar grains perpendicular to the substrate of (0002) crystal orientation, large grain size of 50 ~ 60 nm and small full-width at half-maximum (FWHM) of the X-ray diffraction (XRD) peak of 0.151°; this is comparable to other results obtained from ZnO thin films deposited on rigid substrates^35^,^36^,^37^, demonstrating the crystalline quality of the ZnO is not compromised by being deposited on the PI layer. The details of the characterization results can be found in the SI (Figure S4).

Figure 2c is an optical microscopy image of the PI-FBAR with a 9 μm PI support layer from above. The surface is flat and the device tightly adheres to the rigid Si substrate. This structure is robust, strong and simple, and the yield for the PI-FBARs is extremely high (>95%) if the residual stress in the films is not too high. Figure 2d is an image of the back-trench FBAR after the removal of the back Si. The dark area in the middle of the image is the device on the SiO_2_ membrane, which buckles up due to the residual stress of the ZnO layer^38^. This is a frequently-encountered phenomenon in the fabrication of MEMS devices. When the residual stress is larger than ~1 GPa, the ZnO/SiO_2_ membrane easily breaks, leading to failure of the devices. The yield for the back-trench FBARs is typically 30 ~ 40%, which is less than half that of the PI-FBARs.

Figures 2e and 2f show the reflection and transmission spectra of the devices. The PI-FBAR and back-trench FBAR have resonant frequencies at 1.53 GHz and 1.57 GHz respectively. The signal amplitude and insertion loss of both types of the FBARs are comparable to each other. The quality factor, *Q,* can be estimated by^39^,
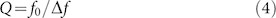
where *f_0_* is the resonant frequency and Δ*f* the bandwidth at −3 dB of the maximum of the resonant peak. The average *Q*-value of the back-trench FBARs is about 493, while that for the FBARs on the PI support layer is about 470, which are comparable to each other. The performance of the two FBAR architectures is similar, and also is comparable to previously-reported results^40^,^41^.

From theoretical analyses and modeling, it is clear that the thickness of the PI support layer should affect the performance of the FBAR significantly. Thus experiments were carried out to investigate the effect of the thickness, *d*, of the PI layer on the performance of the PI-FBAR. Figures 2g and 2h are the corresponding reflection S_11_ and transmission S_12_ spectra of the PI-FBARs with a ZnO thickness of ~2.0 μm and various PI thicknesses. The resonant peak has small amplitude when the PI thickness is thin, and increases with the PI thickness, in agreement with the modeling result as shown in Figure 1d. The structures are the same except for the PI thickness. The resonant frequency was found to be 1.53 ± 0.05 GHz for all the devices; this is within the experimental scatter that would be expected from variation of the ZnO thickness deposited from run to run. The results indicate that the PI thickness has little effect on the resonant frequency of the PI-FBARs, but has a significant effect on the signal amplitude, in agreement with the modeling results.

Figure 2i compares the simulated reflection spectrum of a PI-FBAR with the experimental one with a 9 μm PI layer and a ~2.0 μm ZnO layer. The resonant frequency of the fabricated FBAR is in agreement with the simulated one, but the signal amplitude is much smaller than that of the simulated one. Single crystalline ZnO layer with ideal material properties was used in the simulation, while the practical material is polycrystalline and has a high density of defects, making the waves scatter and attenuate severely, thus the signal amplitude is smaller compared to the ideal case. Figure 2j shows the dependence of the *Q*-factor on the thickness of the PI support layer for the fabricated FBARs. The *Q*-factor increases with the PI thickness initially and then becomes constant at *d* ≥ 9 μm, *i.e.* the critical thickness is smaller than 9 μm, in agreement with the modeling as shown in Figure 1.

### FBARs on arbitrary substrates

**Figure 3:**
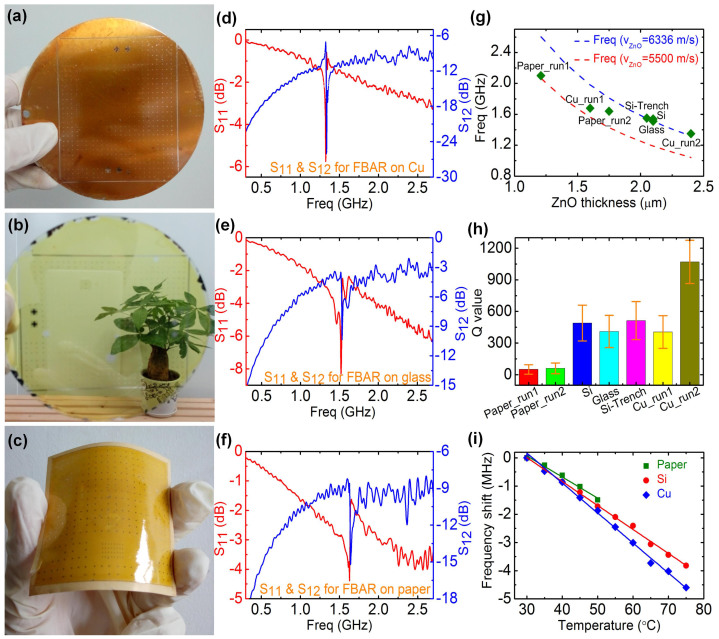
PI-FBARs on different substrates. (a), (b) & (c) Photographs of the PI-FBAR fabricated on copper plate, glass and paper substrates, respectively, showing the transparency and flexibility for the latter two types. (d), (e) & (f) Reflection and transmission spectra of the PI-FBARs fabricated on copper plate, glass and paper substrate respectively. (g) The resonant frequency as a function of ZnO thickness for FBARs on various substrates, and the two dotted lines are calculated with an idea acoustic velocity (6335 m/v) and a low acoustic velocity of 5500 m/s. The frequency decreases monotonically with the increase in ZnO thickness, and approaches the ideal case at thick ZnO of about 2.0 μm. ‘paper_run1’ and ‘paper_run2’, and ‘Cu_run1’ and ‘Cu_run2’ refer to samples made in different runs with similar conditions. (h) Comparison of the *Q*-values for FBARs fabricated on various substrates. The *Q*-values are relatively constant for those on Si and glass substrates, but varies significantly for those on copper and paper substrates, mostly due to the difficulty in controlling the ZnO thickness and quality on these two substrates. (i) Frequency shift as a function of temperature for PI-FBARs on three different substrates, showing a linear relationship for all the PI-FBARs. The *TCF* is −45.47 ppm/k, −63.37 ppm/k, −54.56 ppm/k for the FBARs on paper, copper and Si substrates respectively.

The above results demonstrated that an FBAR architecture using a polymer support layer can be effective, and it provides a means to fabricate FBARs on arbitrary substrates, including flexible substrates or substrates with uneven surfaces. Detailed FEA modeling shows indeed the type of the substrate material has little impact on the performance of the FBARs (Figure S3a), but the type of support layer as shown in Figure S3b due to the difference in acoustic impedance as explained later. To test this hypothesis, FBARs were further fabricated on copper plate, glass and paper as shown in Figures 3a, 3b and 3c, respectively, with the corresponding reflection spectra shown in Figures 3d, 3e and 3f. The papers used for the fabrication of FBARs are the normal laser printer paper with no any treatment. The comparison of the transmission spectra for the FBARs on four types of substrates is shown in Figure S5 in SI. The PI thickness in these devices is 9 μm (*i.e.* above the critical thickness), while the thickness of the ZnO layers varies (unintentionally) for different samples, especially those on copper and paper substrates due to the difficulty in controlling the deposition conditions for these substrates, but overall it does not affect the conclusions as discussed later.

The resonant frequencies for the FBARs on Si (including the trench-type FBARs) and glass substrates are relatively constant at about 1.50 ~ 1.58 GHz, while those for the FBARs on copper and paper substrates vary significantly from run to run (designated as paper_run1, paper_run2, Cu_run1 and Cu_run2). Resonant frequency, *f_r_*, of an FBAR is determined by *f_r_* = *ν*/(2*d*), where *ν* is the acoustic velocity and *d* the thickness of the PE layer. The relationship between *f_r_* and *d* has been investigated in detail for FBARs on various substrates as shown in Figure 3g. The two dotted lines are the resonant frequencies calculated using the ideal acoustic velocity of 6336 m/s^42^ and a low velocity of 5500 m/s. It is clear that the resonant frequency decreases monotonically with the increase in ZnO thickness. However, *f_r_* for the devices with thin ZnO layers is much smaller than the ideal cases, and approaches the ideal case when the ZnO thickness increases. This can be briefly explained by the fact that there is a transitional layer in sputter-deposited ZnO layers which has an amorphous or fine-grain polycrystalline microstructure with a thickness of tens to hundreds of nanometers and a correspondingly low acoustic velocity. The effect of this layer on the acoustic velocity of the whole PE layer would be large when the ZnO is thin, and it is possibly responsible for the resonant frequencies lower than the ideal case observed for the thinner layers.

Similarly, the average *Q*-values for those on Si and glass are relatively constant in the range of 450–550, but vary significantly from 970 to 405 and 51 to 70 for the devices on copper plates and papers of different runs with two run examples shown in Figure 3g. The devices were fabricated by identical conditions, and the scatter of the Q-values is mainly caused by the variation of ZnO thickness and crystal quality on copper and paper substrates, on which the deposition processes are yet to be optimized as they have very different thermal capacities and conductivities from that of Si (or glass). Nevertheless, the results demonstrate that FBARs can be fabricated on any substrate with a polymer support layer, and a *Q*-factor over 500 is achievable if the process is optimized, which is comparable to or better than those reported using traditional architectures^43^,^44^,^45^.

The transmission signal amplitude is small for the FBARs on a paper substrate, partially due to the deterioration and deformation of the paper substrate during the solvent-related processes (see Figure S6 in SI), but it shows a well-defined resonant peak with the *Q*-values in the range of 50–70, demonstrating the feasibility and possibility of fabricating FBARs on soft and flexible substrates. The FBARs on glass are transparent, and the back image can be clearly seen. If Al-doped ZnO (AZO) and indium tin oxide (ITO) are used as the electrodes, then they can be used as transparent devices as demonstrated by our previous transparent surface acoustic wave devices^46^. The summary of the characteristics of the PI-FBARs on various substrates and the back-trench FBAR is shown in Table S1 in SI for clarity.

Figure 3i is the frequency shift as a function of temperature for FBARs on Si, copper and paper substrates. All the frequency decreases linearly with the increase in temperature. The temperature coefficient of frequency (*TCF* = Δ*f*/Δ*T*) can be obtained from the gradients. It is −45.47 ppm/k, −63.37 ppm/k and −54.56 ppm/k for the FBARs on paper, copper and Si substrates respectively. Although the thermal expansion coefficient for paper and copper is much larger than that of Si, the *TCF* does not change significantly, implying the effective decoupling of the FBARs from the substrate.

### FBARs on a substrate with uneven surface

**Figure 4:**
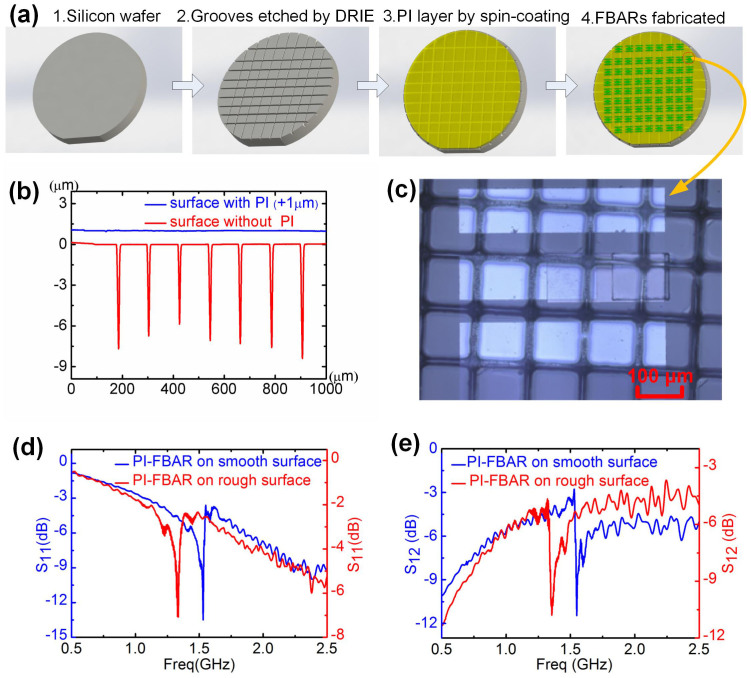
PI-FBARs on a substrate with uneven surface. (a) Process steps used to fabricate the FBARs on a substrate with uneven surface; (b) Comparison of the surface roughness measured by a profilometer. The red line represents the surface with etched trenches that have a width of 10 μm and a depth of ~8 μm, and the blue line represents the roughness of the surface coated with a 9 μm PI layer, showing a very flat and smooth surface; (c) A microscopy image of the fabricated PI-FBAR on a PI-coated uneven Si-wafer; (d) and (e) Comparison of reflection and transmission spectra for the PI-FBARs on smooth surface (blue) and uneven surface (red), showing similar performance to each other.

Integration of FBARs with CMOS is essential, as discussed in the Introduction, but has been severely restricted due to limitations of previous architectures. The surface of modern CMOS chips with multiple interconnects and passivation layers is uneven with steps as high as a few micrometers. Since a polymer layer formed by spin-coating can provide a smooth coating over such a surface, the polymer support layer architecture allows FBAR fabrication on CMOS directly. To demonstrate this, we fabricated PI-FBARs on silicon wafers with deliberately etched deep trenches. A typical surface is shown in Figures 4a and 4c. Figure 4b provides a comparison between the surface roughness before and after spin-coating of a 9 μm PI layer over the trenches etched by DRIE that have a width of 10 μm and depth of about 8 μm. Figures 4d and 4e compare the reflection and transmission spectra for the PI-FBARs on the uneven and flat Si wafers. The spectra of both the FBARs show similar performance with the average *Q*-values ~460. The shift of the resonant frequency is mainly due to the variation of the ZnO thickness between processing runs, during which the equipment and process conditions may have changed after many experiments by others for other materials in the laboratory. The resonant frequency and Q-value of FBARs can be easily controlled if manufactory conditions can be used.

## Discussion

The theoretical analysis and FEA modeling demonstrate that a resonator structure on a polymer support layer with low acoustic impedance and thickness above a critical value is a good FBAR architecture. Acoustic waves do enter the polymer support layer, but are quickly attenuated. To make high performance FBARs, the key is to select materials for the bottom electrode and the support layer with large and small acoustic impedance respectively.

For the support layer, many polymers with low *Z* are available. We have studied the effect of polymer support layer with different acoustic impedances with the results shown in Figure S3. The results show that if the polymers have similar acoustic impedances, *e.g.* PI and SU8, then a similar thickness of the polymers is needed to achieve decoupling of the FBARs from the substrate; when the acoustic impedance of the polymer support layer is small such as for polydimethylsiloxane (PDMS), the thickness of the support layer can be greatly reduced as shown by Figure S3b. However PDMS has lower processing temperatures (<100°C), and it is difficult to deposit a high quality PE layer on top of it. If the PE layer can be deposited at room temperature using special techniques like high target utilization sputtering (HiTUS) at room temperature^8^,^12^,^34^, then PDMS would be the ideal polymer support layer for the fabrication of FBARs. On the other hand, many acoustic absorber materials have been developed using porous structures such as sol-gels and foams^33^, or acoustic metamaterials^47^, and it is expected these would be very effective support layers on solid substrates for the fabrication of FBARs on a support layer.

Nevertheless, the results clearly demonstrated that FBARs can be fabricated on arbitrary substrates with performance comparable to those made by traditional technologies, and the general feasibility of flexible and transparent FBARs has also been shown. The new architecture offers many advantages over the existing architectures. First, the FBAR on the polymer support layer allows the fabrication of high performance devices on arbitrary substrates (such as flexible and transparent polymer, rigid and non-planar metal foils and semiconductor wafers) of almost any surface morphology. This also allows constructing 3D stacked electronics. The versatile substrate selection could greatly broaden resonator applications in advanced electronics such as flexible, wearable or epidermal electronics. Second, the fabrication process is very simple, and the yield is very high. Furthermore, the device structure is very simple, and therefore the devices are extremely robust and strong.

In conclusion, a new FBAR architecture in which the resonant structure is on a polymer support layer with low acoustic impedance and high attenuation coefficient has been proposed, and verified by finite element analysis and experiments. Both approaches confirmed that a PI layer with a thickness ≥9 μm produces high performance FBARs operating at 1.3–2.5 GHz. The FBARs show high transmission signal and high quality factor typically at ~500, comparable to those obtained by other FBAR architectures. The PI-FBAR devices on solid substrate are robust, strong, and the fabrication process is very simple with the yield up to 95% in laboratory conditions, which is much higher than the other architectures. Feasibility for the integration of FBARs with CMOS on the same substrate has also been demonstrated. Therefore the polymer support FBAR architecture will enable new applications of this technology.

## Methods

### FBAR device modeling

Finite element analysis was used to model the structural deformations and the corresponding reflection characteristics of the FBAR structures using the COMSOL Multiphysics software. The material properties of the polyimide are taken from the maker's data sheet and the ZnO layer from Ref. 27, and the Young's modulus, Poisson's ratio and density were set to be 70 GPa, 0.35 and 2.7 g/cm^3^ for Al, for the modeling.

### The PI support layer formation

The formation of a good PI layer is the key to making high performance PI-FBARs. Polyimide resin (ZKPI-305IIE, POMESci-techCo. Ltd. Beijing) was used. Before coating, the substrate (not paper) was thoroughly cleaned using acetone, IPA and deionized (DI) water, and dried using N_2_ gas. For better adhesion, the substrate was baked at 100°C for 10 min to remove moisture. PI resin was then dropped on the surface of the substrate and spun at various speed for 60 s to achieve different thickness support layers. It was then baked on a hot plat at ~240°C for 3 hr. For a spin speed of 1000 RPM, the thickness of the PI layer was ~9 μm, and the surface rough was in the range of 55 ± 5 nm.

### PI-FBAR device fabrication

PI-FBAR devices were fabricated by a three-mask process: bottom electrode formation, etch of vias and top electrode formation. Whereas the trench-type FBARs were fabricated by a process similar to that for the PI-FBARs with an additional back trench etching process. The detailed process is as follows.

The bottom electrodes were formed by a UV-light photolithography and lift-off process. Positive photoresist (AR-P 5350) was used for patterning the electrodes on the top of the PI layer, while Al (~100 nm) was deposited by thermal evaporation. Afterward, ZnO piezoelectric films were deposited using a direct current (DC) reactive magnetron sputtering system. The deposition process has been optimized before for Si and glass substrates^46^ and used here without further optimization. The base pressure of the chamber was 1 × 10^−4^ Pa before deposition. A water-cooling zinc target of purity 99.999% (100 mm diameter and 3 mm thickness) was used for the deposition of the ZnO films with a 70 mm distance from the substrate. The deposition conditions were 200°C substrate temperature, 1 Pa deposition pressure, O_2_/Ar gas mixture at a ratio of 50/100 sccm, 200 W sputtering power, and −75 V bias voltage. We targeted FBARs with a ZnO thickness of 2.0 μm, but this varied slightly from wafer to wafer, leading to variation of resonant frequency of the FBARs.

After ZnO deposition, vias through the ZnO for electrical connection to the Al bottom electrodes were formed by UV photolithography, and etched in a 1% hydrochloric acid solution at room temperature to remove the ZnO in the vias. The top electrodes were then formed by photolithography and lift-off process with identical materials and thicknesses to the bottom electrodes.

For etching the back trench of trench-type FBARs and deep trenches for the uneven surface experiments, a deep reactive ion etch (DRIE) system (Plasmalab System 100) was used. The plasma power was 800 W. The etching time was approximately 300 min for the back-trenches, and 5 min for the deep trenches. All the devices have an active area of 100 × 200 μm^2^.

### Material and device characterizations

ZnO crystalline structure and device structure were analyzed by scanning electron microscope (SEM) (Hitachi S-4800) with an accelerating voltage of 5 *k*eV. The surface roughness of the films and profile of the structure were investigated by atomic force microscopy (AFM) (SPI-3800N, Seiko Co.) and profilometer (KLA-Tencer, D-100). The transmission properties of the FBAR devices were measured using an Agilent E5071C network analyzer.

## Author Contributions

X.W., H.J., S.D., S.L. and J.L. conceived of the project, and designed the experiments. G.C. conducted the experiments. X.Z. conducted the numerical simulation. G.C., X.W., S.D., J.L., A.F. and W.I.M. wrote the paper. All authors discussed the results and commented on the manuscript.

## Supplementary Material

Supplementary Information Supplementary information
