# Supplementary material for: Film bulk acoustic resonators integrated on arbitrary substrates using a polymer support layer
Source: Sci Rep. 2015 Mar 31;5:9510. doi: 10.1038/srep09510 (PMC4379503; doi:10.1038/srep09510)
Supplement: Supplementary Information [file srep09510-s1.pdf]

Supplementary Information

Film bulk acoustic resonators integrated on arbitrary substrates using a polymer support layer

Guohao Chen[1], Xinru Zhao[1], Xiaozhi Wang[1*], Hao Jin[1], Shijian Li[2], Shurong Dong[1], A.J. Flewitt[3], W.I. Milne and J.K. Luo[4,1*]

[1] Department of Information Science and Electronic Engineering, Zhejiang University and Cyrus Tang Center for Sensor Materials and Applications, Zhejiang University, Hangzhou 310027,China.

[2] College of Computer Science, Zhejiang University, Hangzhou, 310027, China

[3] Electrical Engineering Division, University of Cambridge, J J Thomson Avenue, Cambridge, CB3 0FA U.K.

[4] Institute of Renewable Energy and Environmental Technologies, Bolton University, Deane Road, Bolton BL3 5AB, UK.

*Correspondence and requests for materials should be addressed to X.W.(email: xw224@zju.edu.cn) or J.L. (email:J.Luo@bolton.ac.uk).

1. Theoretical analysis and modeling

Consider a film bulk acoustic resonator (FBAR) consisting of a piezoelectric (PE) layer sandwiched by two metal electrodes, sitting on a polymer support layer on a substrate as shown in Figure 1a. We assume that the bottom electrode has similar properties to those of the PE layer, and it can be considered as part of the (PE) layer, thus the FBAR structure can be simplified as shown in Figure S1a for easy theoretical analysis. Standing plane waves are generated between the two electrodes under RF excitation. At the interface between the PE layer and the polymer support layer, the waves will partially transmit and reflect at the interface with the support polymer, and this is discussed in detail in the main article.

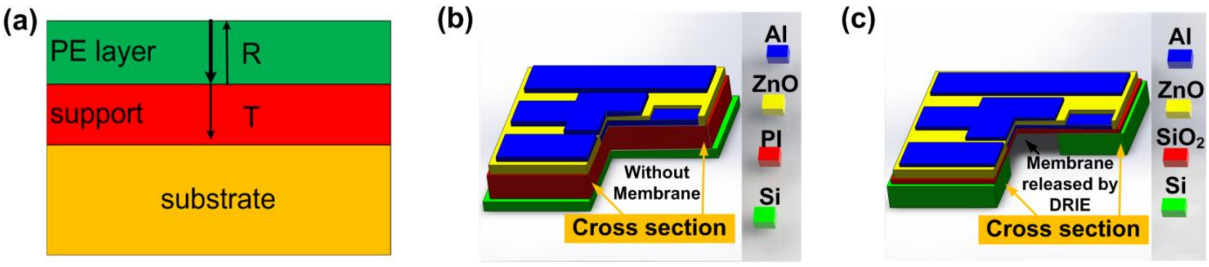

Figure S1. (a) Schematic structure of the proposed PI-FBAR and (b) the simplified multilayer structure for the FBAR. The plane waves partially transmit through the interface between the PE and support layer. The two electrodes on both sides of the active FBAR area form a coplanar wave guide to minimize the interference of the environment.

Figure S1b and S1c are 3-dimensional (3D) schematics of the new architecture FBAR on a polyimide (PI) support layer (designated as PI-FBAR in the main article) and the back-trench FBAR (the membrane architecture as mentioned in the article), respectively, showing the details of the device structures. The PI-FBAR is solidly mounted on a polymer support layer, thus it is robust and strong, and very simple in structure and easy to fabricate, leading to high throughput and yield better than the trench-type FBARs.

Figure S2 shows the evolution of displacements in 2D and 3D of the layers in the new FBAR architecture with varying PI thickness with the ZnO PE layer sandwiched by two Al electrodes. The structure used in the finite element analysis (FEA) modelling is the same as that shown in Figure S1b. A Si substrate of 20 μm, Al electrodes of 100 nm and a ZnO layer of 2.0 μm were used in the modelling. For the FBAR structure directly on the Si substrate, the displacement amplitudes in the PE and the Si layers are identical and there is little acoustic wave attenuation in the Si as the Si bulk is assumed to be a perfect crystalline material with no acoustic scattering and thermal absorption. The displacement amplitude in the PE layer increases and saturates, while that in the Si substrate decreases with increase in the PI thickness, and disappears at a PI thickness about 9 μm, in agreement with the theoretical analysis discussed in the article. In this case, the acoustic wave entering the polymer layer diminishes exponentially within the PI layer and does not transmit the acoustic energy to the elastic Si substrate, enhancing the performance of the FBAR device.

By using a polymer support layer, the FBAR is effectively decoupled from the substrate, therefore the PI-FBARs can be fabricated on arbitrary substrates without being affected by the material properties of the substrate. Figure S3a provides a comparison between the simulated displacements in each layer of the FBARs made on PI, Si and copper substrates, showing similar results for the FBARs on Si and copper substrates though that produced directly on a PI film shows a somewhat different behaviour.

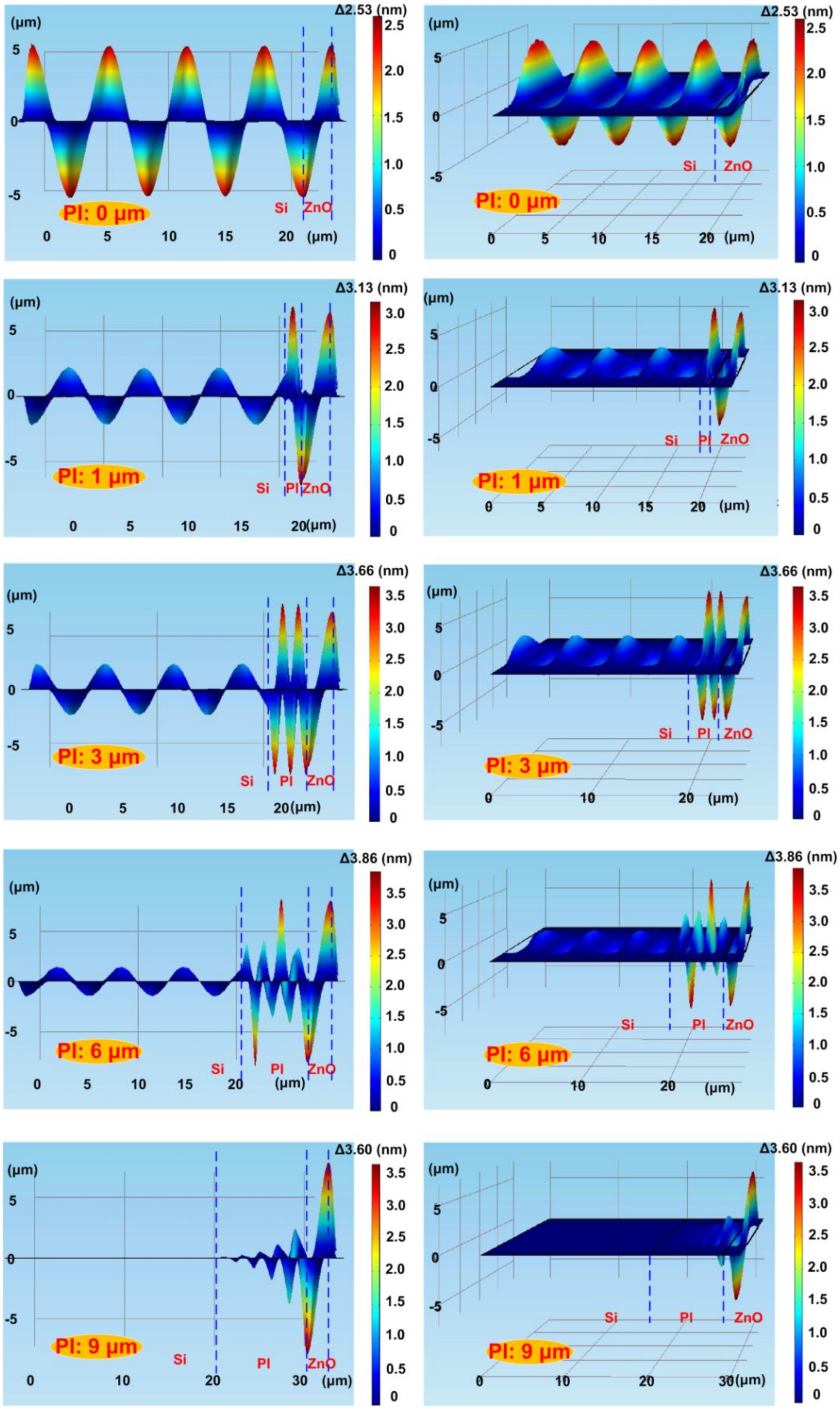

Figure S2. Evolution of displacement in the FBAR structure with varying the PI layer thickness. As the PI layer becomes thicker, the displacement in the PE layer increases whist that in the Si disappears when the PI layer is about 9 μm. A Si substrate of 20 μm, Al electrodes of 100 nm and a ZnO layer of 2 μm were used in the modelling. Note Figure S1a is not based on an exact 2D figure, but rather viewed from a wide angle towards to narrow one.

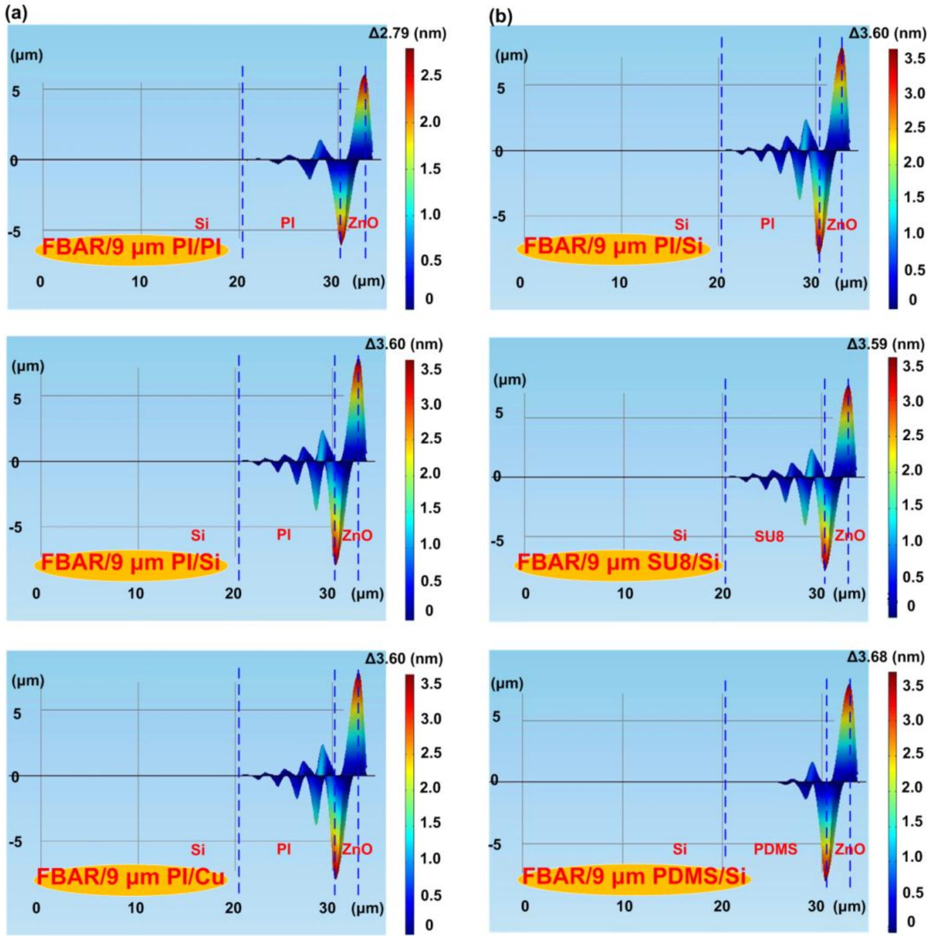

Figure S3. (a) Comparison of displacements in FBARs made on PI, Si and copper substrates and (b) in FBARs with different types of polymer support layers. FBARs made on Si and copper substrates show similar displacements in each layer under RF excitation, indicating that the FBARs are effectively decoupled from the substrate, and will not be affected by the material properties of the substrate. The FBAR on a PI film shows some different behaviour which remains to be investigated in detail. FBARs wi th SU8 or PI support layer show similar behaviour as both the polymers have similar acoustic impedances, whilst that on a PDMS support shows a much shorter wave attenuation distance as it has near-zero acoustic impedance.

On the other hand, according to Figure 1b in the article, the properties of the materials for the bottom electrode and the support layer are very important in obtaining high performance FBARs. The best materials for the bottom electrode are those with high acoustic impedance and low mass density such as Cr and Mo, while polymers such as polydimethylsiloxane (PDMS), poly(methyl methacrylate) (PMMA) and PI are suitable as the support layer. Figure S3b provides a comparison of the displacements in each layer of the FBARs with different types of polymer support layer. The wave attenuation distance in the FBAR on a SU8 support layer is similar to that in the PI-FBAR as both the polymers have a

similar acoustic impedance as shown in Figure 1b in the article, while that in the FBAR on a PDMS support layer is much shorter as it has a near-zero acoustic impedance. This implies that a very thin PDMS layer could be used as the polymer support for the fabrication of high performance FBARs (if the thermal budget issue can be solved).

2. Crystal characterization

The ZnO crystal structure was characterized by scanning electron microscopy (SEM), atomic force microscopy (AFM) and X-ray diffraction (XRD). Figure S4a shows an SEM image of the cross section of a typical ZnO film with a thikcness of ~2 μm. The ZnO film consists of columnar nanograins perpendicular to the substrate. XRD curve reveals a single strong peak which corresponds to the (0002) crystal orientation of ZnO crystal (Figure S4b). The full-width at half-maximum (FWHM) of the XRD peak is 0.151 °, comparable to most of the results obtained from ZnO thin films deposited on rigid substrates. The roughness measured by atomic force microscopy is in the range of 8-12 nm for the ZnO film thickness of about 2 μm. All these demonstrate the high crystalline quality of the thin films deposited on the PI layer.

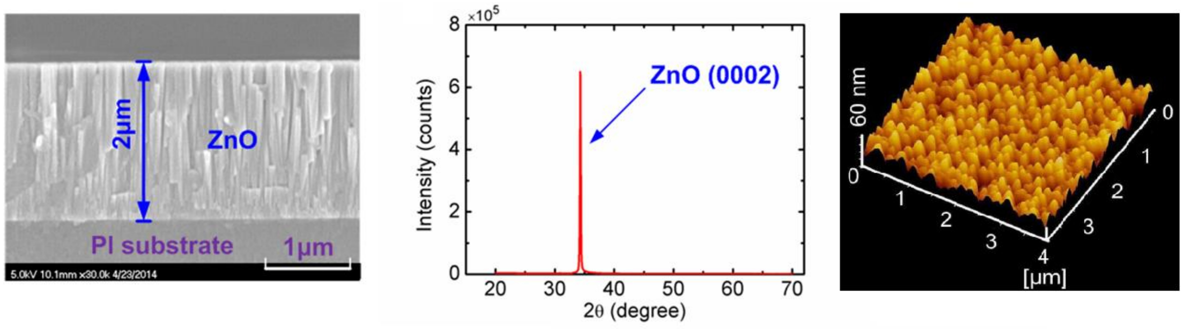

Figure S4. (a) An SEM image of the cross-section and (b) the XRD pattern of the ZnO layer used for the fabrication of FBARs. Both results show that the ZnO has a (0002) crystal orientation with large grain size. (c) is an AFM image showing the surface roughness of a 2 μm ZnO film, which is in the range between 8-12 nm.

3. FBAR device characterization

Figure S5 provides a comparison of transmission spectra for FBARs made on various substrates. The resonant frequency and signal amplitude for the FBARs on Si and glass are very close, while those on copper and paper substrates are quite different. The different resonant frequencies, especially those on copper and paper are mainly due to the different

ZnO PE layer thicknesses deposited at different times as discussed in the main article. The ZnO deposition process was mainly optimized using a Si substrate, whereas the thermal capacity and conductivity of copper and paper substrates are very different from those of Si, making the deposition processes slightly different from that on Si and glass substrates. The detailed characteristics of the FBARs on various substrates are summarized in Table S1. The FBARs on Si (includes the trench-type FBAR) and glass substrates have similar characteristics, while those on copper and paper substrates have very different ones.

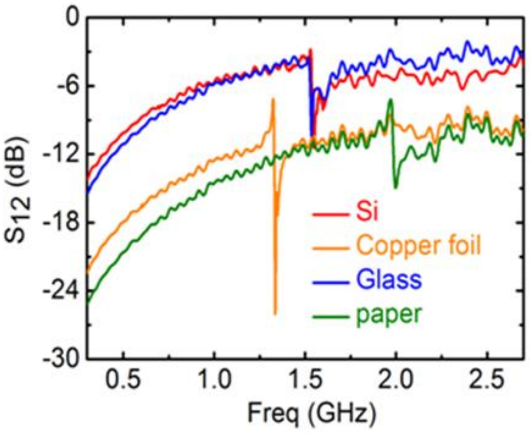

Figure S5. Comparison of transmission properties of FBARs made on various substrates.

Table S1 Summary of characteristics for the PI-FBARs on various substrates and trench-FBAR.

	**Substrate**	**Density (kg/cm^3)**	**ZnOthickness (μm)**	***E* (GPa)**	**f_r (GHz)**	**Amplitude (dB)**	**Loss (-dB)**	***Ave. Q factor***
**PI-FBAR**	***Paper_1***	1.49	1.21	5.0-10	2.00	7.65	14.94	51
	Paper_2	1.49	1.75	5.0-10	1.64	6.46	15.25	70
	Si	2.33	2.10	131	1.54	8.67	11.31	470
	Glass	2.39	2.11	69.3	1.53	7.70	10.29	410
	Cu_1	8.96	1.60	73.8	1.68	10.74	15.84	405
	Cu_2	8.96	2.40	73.8	1.33	18.85	26.06	970
**Trench FBAR**			2.05		1.56	7.91	13.31	493

Figure S6 shows microphotos of the FBARs on a paper substrate, showing the rough surface which was caused by non-uniform swelling during the solvent-based process. The uneven surface breaks or damages the ZnO film, deteriorating the performance of the PI-FBARs on a paper substrate.

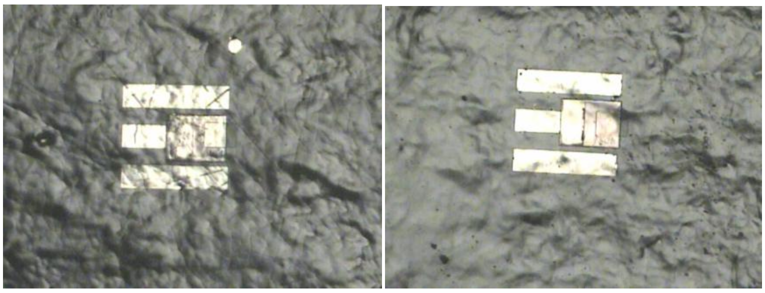

Figure S6. Microphotos of the FBARs on a paper substrate, showing the long-range rough surface which is mainly caused by the deformation (swelling) of the paper in solution during the photolithography process.
